# Depressive Symptoms, Insomnia-Related Difficulties and Self-Rated Health Among Community-Dwelling Adults Aged 40 Years and Older in Western China: A Cross-Sectional Structural Equation Modelling Study

**DOI:** 10.3390/healthcare14203528

**Published:** 2026-10-20

**Authors:** Lansicheng Yao, Jacquline Tham

**Affiliations:** 1 Foreign Affairs Office, North Sichuan Medical College, Nanchong 637000, China; 012024070521@gsm.msu.edu.my; 2 Faculty of Business Management and Professional Studies, Graduate School of Management, Management and Science University, Shah Alam 40100, Selangor, Malaysia

**Keywords:** self-rated health, depressive symptoms, insomnia severity, medical help-seeking attitudes, neighbourhood built environment, family functioning, structural equation modelling

## Abstract

**Highlights:**

**What are the main findings?**

• Fewer insomnia-related difficulties were associated with better self-rated health, with the largest standardised direct coefficient in the model (reverse-coded ISI: β = 0.383, *p* < 0.001).
• Only depressive symptoms showed a supported statistical indirect association with self-rated health through the reverse-coded ISI construct (estimate = 0.208, 95% CI: 0.134–0.294).

**What are the implications of the main findings?**

• The findings support considering depressive symptoms and insomnia-related difficulties together when investigating perceived health among community-dwelling adults.
• The supported indirect association was specific to depressive symptoms, rather than shared across all proposed predictors.

**Abstract:**

Background/Objectives: This study examined the associations of depressive symptoms, medical help-seeking attitudes and perceived neighbourhood built environment with self-rated health, including statistical indirect associations through an Insomnia Severity Index (ISI)-derived sleep construct, among community-dwelling adults in western China. Methods: This secondary cross-sectional analysis included 433 adults aged ≥40 years in Gaoping District, Nanchong, China. Self-rated health was assessed using the SF-36 General Health subscale. PHQ-9 and ISI indicators were reverse-coded so that higher values represented lower depressive symptom burden and fewer insomnia-related difficulties, respectively. Structural equation modelling estimated direct and indirect associations, with 5000 bootstrap resamples. Multi-group analysis compared the ISI–self-rated health path between family-functioning groups. Results: Model fit indices were CFI = 0.955, TLI = 0.953 and RMSEA = 0.037. The ISI-derived construct had the largest standardised direct coefficient for self-rated health (β = 0.383, *p* < 0.001). Reverse-coded PHQ-9 was associated with ISI (β = 0.544, *p* < 0.001) and self-rated health (β = 0.164, *p* = 0.009). Medical help-seeking attitudes were directly associated with self-rated health (β = 0.193, *p* = 0.001). The PHQ-9–ISI–self-rated health indirect estimate was 0.208 (95% CI, 0.134–0.294). Corresponding indirect associations for help-seeking attitudes and neighbourhood environment were not supported. NEWS-A direct paths and the family-functioning group difference were not statistically significant. Conclusions: The findings supported an ISI-related statistical indirect association between depressive symptoms and self-rated health within the specified model. They do not establish causal mediation or intervention effectiveness.

## 1. Introduction

Self-rated health (SRH) is a widely used indicator of perceived health, functional vulnerability and future health risk [1]. SRH has been conceptualised as a global health appraisal that integrates physical symptoms, psychological state, functional capacity, social context, and expectations about future health [2,3,4,5]. Evidence from Chinese middle-aged and older adults has shown that fair or poor SRH is associated with increased all-cause mortality, supporting its value as a low-burden marker for population health assessment [2]. As global and Chinese populations continue to age, scalable indicators such as SRH are increasingly important for community health management. The World Health Organization projects that the global population aged 60 years and above will reach 2.1 billion by 2050 [5,6], and China reported 323.38 million people aged 60 years and above at the end of 2025, accounting for 23.0% of the national population [7].

Adults aged 40 years and above represent a transitional population in which psychological burden, sleep problems, chronic disease risk, family responsibility, and healthcare needs may begin to accumulate [8]. This is particularly relevant in western China, where community health management may need to identify perceived health vulnerability before severe functional decline or avoidable healthcare use occurs [7,9]. Although depressive symptoms, medical help-seeking attitudes, sleep quality, family functioning, and neighbourhood environment have each been linked to SRH, their relative roles remain insufficiently understood when these factors coexist in the same community population.

This study was guided by an integrated psychosocial–behavioural–environmental framework of SRH, grounded in the social determinants of health perspective [10,11]. Within this framework, depressive symptoms were specified as a psychosocial correlate of perceived health [12]; medical help-seeking attitudes reflect behavioural readiness to respond to health concerns [12]; and perceived neighbourhood built environment represents an environmental correlate related to access, walkability, safety, and neighbourhood resources [13]. Considering these domains together enables the relative associations of psychological, behavioural, and environmental factors with SRH to be estimated within one model.

Sleep quality is relevant to this framework because it represents a proximal health-related experience through which psychological burden may be reflected in fatigue, somatic discomfort, reduced daily functioning, and poorer perceived health [14,15]. Recent evidence from Chinese older adults has shown that poor sleep quality is common and is more pronounced among individuals with depressive symptoms and chronic conditions, highlighting the relevance of sleep assessment in ageing-related community health research [16]. However, whether sleep quality serves as a statistical pathway linking depressive symptoms and SRH after behavioural and environmental correlates are considered remains unclear.

Accordingly, this study examined an integrated psychosocial–behavioural–environmental model of SRH among community-dwelling adults aged 40 years and above in western China. Depressive symptoms, medical help-seeking behaviour, and perceived community-built environment were specified as three theoretically distinct correlates. Sleep quality was examined as a proximal statistical pathway linking these correlates, particularly depressive symptoms, to SRH, while family functioning was examined as a contextual moderator of the sleep quality–SRH association.

## 2. Materials and Methods

### 2.1. Study Design and Participants

This study was a secondary cross-sectional analysis of data collected during a community health survey conducted in July and August 2023 in Gaoping District, Nanchong City, Sichuan Province, China. The parent survey covered three townships and two urban subdistricts. Multistage cluster sampling was used, with random selection of village or neighbourhood committees, followed by household sampling within the selected communities. One permanent resident per household was selected using the nearest-birthday method.

Participants were eligible if they were aged 40 years or older, had resided in the community for at least six months, could complete the questionnaire independently or with assistance, and provided informed consent. Exclusion criteria included severe physical or mental illness that prevented reliable participation, being bedridden or institutionalised, acute illness during the survey period, and an inability to communicate effectively.

The present analysis examined depressive symptoms, medical help-seeking attitudes, perceived neighbourhood built environment, sleep quality, perceived family functioning and self-rated health within the same analytical sample. Other analyses of the parent survey have been reported previously; the present study focused on the direct associations of the psychosocial, behavioural and environmental constructs with self-rated health and their statistical indirect associations through sleep quality.

### 2.2. Questionnaire Administration and Data Screening

The questionnaires were administered electronically in Chinese. Trained interviewers administered the questionnaires at community survey sites or at participants’ residences when attendance at a survey site was impractical. Participants completed the questionnaire independently where possible. For those requiring assistance because of limited literacy, visual difficulty or physical limitations, interviewers read the questions aloud and recorded the responses without interpreting the questions or suggesting answers. Interviewer training covered standardised administration, neutrality and participant privacy.

Completed questionnaires were checked for completeness and reviewed for data quality. Screening identified duplicate submissions, invalid questionnaires and straight-line response patterns across the questionnaire. Thirty-four returned questionnaires were excluded, leaving 433 records for analysis. Measurement instruments and scoring procedures are described in Table S1, and data-screening and diagnostic results are reported in Table S5.

Range and logical consistency checks were conducted during data cleaning. Identifying information was removed before analysis, and numerical identifiers were used for data management.

### 2.3. Measures and Score Direction

The measured constructs were represented by their respective questionnaire indicators. Indicator coding was harmonised for the structural model so that higher values represented more favourable states or attitudes. The construct names were retained regardless of scoring direction. In particular, reverse coding of PHQ-9 indicators represented lower depressive symptom burden, rather than a broader construct of psychological health. Similarly, reverse coding of ISI indicators represented fewer insomnia-related difficulties.

#### 2.3.1. Self-Rated Health

Self-rated health was assessed using the five-item General Health subscale of the 36-Item Short Form Health Survey (SF-36 GH). The construct therefore represented perceived general health assessed across five items, rather than a single global self-rated health question. Indicators were oriented so that higher values represented better perceived health. Cronbach’s alpha was 0.874 in the analytical sample.

#### 2.3.2. Depressive Symptoms

Depressive symptoms were assessed using the nine-item Patient Health Questionnaire (PHQ-9) [17,18]. The instrument assesses the frequency of depressive symptoms during the preceding two weeks, with conventional item scores ranging from 0 to 3. For the structural model, the indicators were reverse-coded so that higher values represented lower depressive symptom burden. Previous research has supported the reliability and validity of the Chinese-language PHQ-9 in community residents [19]. The construct was labelled depressive symptoms throughout this manuscript. Cronbach’s alpha was 0.917.

#### 2.3.3. Medical Help-Seeking Attitudes

Medical help-seeking attitudes were assessed using a 10-item adapted measure informed by the professional help-seeking framework of Fischer and Turner [20,21]. Items were rated on a five-point response scale and coded so that higher values represented more favourable attitudes towards seeking medical care. The measure assessed attitudes rather than observed healthcare utilisation. Cronbach’s alpha was 0.931.

#### 2.3.4. Perceived Neighbourhood Built Environment

Perceived neighbourhood built environment was assessed using a 17-item measure based on the abbreviated Neighbourhood Environment Walkability Scale (NEWS-A) [22]. The items covered four domains: access to services, road conditions, natural environment and traffic safety, and public safety. Higher harmonised scores represented more favourable environmental perceptions. In the measurement model, these four domains were represented as first-order factors loading onto a higher-order neighbourhood environment factor. Cronbach’s alpha for the measure was 0.940.

#### 2.3.5. Sleep Quality

The sleep construct was assessed using the seven-item Insomnia Severity Index (ISI) [23]. The ISI measures difficulties initiating and maintaining sleep, early awakening, dissatisfaction with sleep, and the perceived impact and distress associated with these difficulties. Conventional item scores range from 0 to 4, with higher scores indicating greater insomnia severity.

The ISI provided an insomnia-focused measure for examining the proposed sleep-related pathway. ISI indicators were reverse-coded for the structural model so that higher values represented fewer insomnia-related difficulties. Throughout this article, sleep quality refers specifically to this ISI-derived construct and not to a comprehensive assessment of all dimensions of sleep quality. Cronbach’s alpha was 0.912.

#### 2.3.6. Perceived Family Functioning

Perceived family functioning was assessed using the 12-item measure identified in the Supplementary Materials as the Family Assessment Device (FAD) [24,25]. Items were rated on a four-point response scale and coded to represent a consistent direction of family functioning. Cronbach’s alpha was 0.935.

### 2.4. Sample Size

The sample size for this secondary analysis was determined by the availability of eligible records from the parent survey. No separate a priori power calculation was performed for the structural equation model. The analysis included 433 participants. This sample size should not be interpreted as establishing adequate power for every direct, indirect or between-group association. In particular, small indirect associations and differences between family-functioning groups may have been estimated with limited precision.

### 2.5. Statistical Analysis

Analyses were performed using IBM SPSS Statistics version 25.0 and IBM SPSS Amos version 25.0 (IBM Corp., Armonk, NY, USA). Descriptive statistics summarised participant characteristics and construct scores. Pearson’s correlations were used to examine bivariate associations, and internal consistency was assessed using Cronbach’s alpha [26]. Structural equation modelling was used to estimate direct and statistical indirect associations [27,28].

The structural model included direct paths from depressive symptoms, medical help-seeking attitudes and perceived neighbourhood built environment to the ISI-derived sleep construct and self-rated health, together with the ISI–self-rated health path. Model fit was assessed using CMIN/DF, RMR, GFI, AGFI, IFI, TLI, CFI and RMSEA [29]. Indirect associations were estimated using 5000 bootstrap resamples, with 95% confidence intervals excluding zero interpreted as evidence of a statistical indirect association [30,31].

Multi-group SEM was used to compare the ISI–self-rated health path between groups defined by perceived family functioning. Higher directionally harmonised FAD scores indicated better perceived family functioning. A sample-based threshold of 2.00, corresponding to the sample median, defined the lower-functioning group (scores ≤ 2.00; *n* = 265) and the higher-functioning group (scores > 2.00; *n* = 168). This threshold was used for the statistical comparison and was not interpreted as a validated clinical cut-off.

The same structural specification was fitted in both groups. The ISI–self-rated health path was estimated freely across groups and then constrained to equality. The models were compared using a chi-square difference test with one degree of freedom. The comparison evaluated the between-group difference in this path rather than differences in its statistical significance within each group. Measurement invariance across the family-functioning groups was not formally tested; consequently, cross-group measurement comparability was not established, and the path comparison was interpreted cautiously.

### 2.6. Conceptual Framework

**Figure 1:**
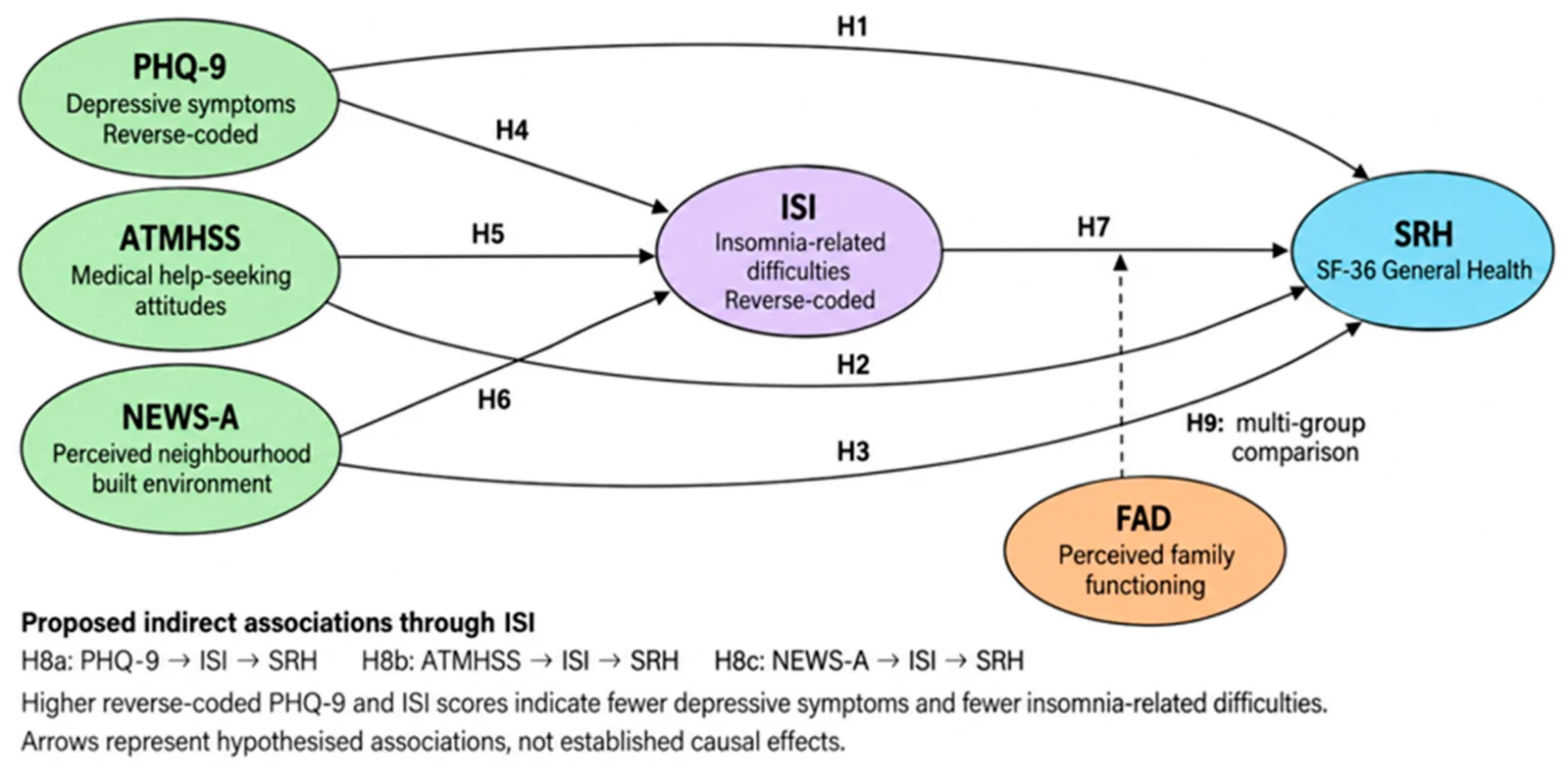
**Conceptual framework.** The dashed arrow indicates the proposed comparison of the ISI–SRH association between groups defined by perceived family functioning (H9).

The conceptual framework comprised depressive symptoms, medical help-seeking attitudes and perceived neighbourhood built environment as predictors of the ISI-derived sleep construct and self-rated health (Figure 1). The model included direct paths from each predictor to both constructs and a path from the ISI-derived construct to self-rated health, allowing three statistical indirect associations to be estimated. Perceived family functioning was used to define groups for comparison of the ISI–self-rated health path. The directional paths represented the proposed analytical structure and did not establish temporal ordering.

### 2.7. Ethical Approval

This study was conducted in accordance with the Declaration of Helsinki. Ethical approval was obtained from the Institutional Ethics Review Board of North Sichuan Medical College, China (approval No. 2024019), and the Ethics Committee of Management and Science University, Malaysia. All participants provided electronic informed consent before participation by selecting “Yes, I have read and agreed to participate” on the first page of the questionnaire.

## 3. Results

### 3.1. Participant Flow and Characteristics

Of the 512 questionnaires distributed, 467 were returned, giving a questionnaire return rate of 91.2% (467/512). Following data-quality screening, 34 questionnaires were excluded and 433 were retained for analysis. Usable questionnaires represented 92.7% of returned questionnaires (433/467) and 84.6% of distributed questionnaires (433/512).

The analytical sample included 260 women (60.0%) and 173 men (40.0%). Participants aged 40–55 years accounted for 66.1% of the sample (*n* = 286). Most participants were married (89.4%), and 47.1% reported high-school, vocational or college-level education. Household economic status was most commonly rated as average (68.6%). Participant characteristics are summarised in Table 1.

### 3.2. Descriptive Statistics and Correlations

Table 2 presents the descriptive statistics and correlations for the harmonised construct scores. Higher values represented more favourable states or attitudes; for PHQ-9 and ISI, this corresponded to lower depressive symptom burden and fewer insomnia-related difficulties, respectively.

All reported correlations were positive and statistically significant at *p* < 0.01. Self-rated health was correlated with the reverse-coded ISI score (r = 0.501), reverse-coded PHQ-9 score (r = 0.463), medical help-seeking attitudes (r = 0.417), perceived neighbourhood built environment (r = 0.325) and perceived family functioning (r = 0.266). The correlation between reverse-coded PHQ-9 and ISI scores was 0.516.

### 3.3. Measurement and Structural Model Fit

**Figure 2:**
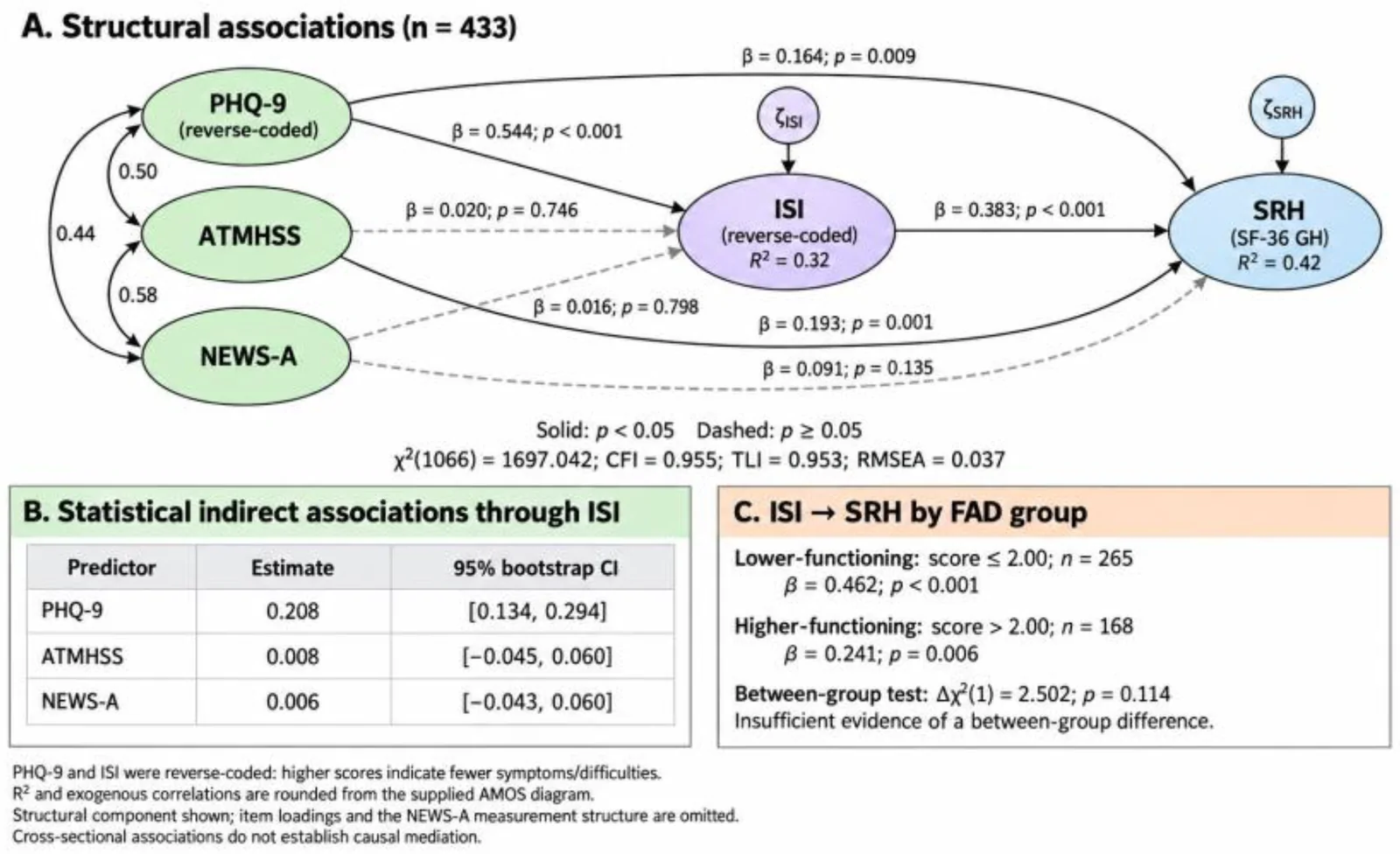
Structural model estimates and family-functioning group comparison.

The measurement model yielded a chi-square value of 2466.554 with 1691 degrees of freedom (χ^2^/df = 1.459), CFI = 0.955, TLI = 0.953 and RMSEA = 0.033. Measurement-model fit, standardised factor loadings, composite reliability, average variance extracted and discriminant validity are reported in Tables S2–S4. The overall measurement model is shown in Figure S7. Figure 2 summarises the structural associations, and the full item-level structural model is provided in Figure S8.

For the structural model, χ^2^ was 1697.042 with 1066 degrees of freedom (χ^2^/df = 1.592). The CFI was 0.955, TLI was 0.953 and RMSEA was 0.037. Additional fit indices are reported in Table 3. The model accounted for approximately 32% of the variance in the ISI-derived construct and 42% of the variance in self-rated health.

### 3.4. Direct Associations

Higher reverse-coded PHQ-9 scores were associated with higher reverse-coded ISI scores (β = 0.544, *p* < 0.001), indicating an association between lower depressive symptom burden and fewer insomnia-related difficulties. The ATMHSS–ISI and NEWS-A–ISI paths were not statistically significant (β = 0.020, *p* = 0.746 and β = 0.016, *p* = 0.798, respectively).

Within the specified model, a one-standard-deviation higher reverse-coded ISI latent score was associated with a 0.383-standard-deviation higher self-rated health latent score, conditional on the other modelled predictors. The reverse-coded ISI construct had the largest standardised direct coefficient for self-rated health among the predictors included in the model (β = 0.383, *p* < 0.001). Medical help-seeking attitudes (β = 0.193, *p* = 0.001) and the reverse-coded PHQ-9 construct (β = 0.164, *p* = 0.009) were also positively associated with self-rated health. The NEWS-A–self-rated health path was not statistically significant (β = 0.091, *p* = 0.135). Standardised and unstandardised estimates are presented in Table 4.

### 3.5. Statistical Indirect Associations Through ISI

The estimated indirect association between the reverse-coded PHQ-9 construct and self-rated health through the reverse-coded ISI construct was 0.208 (95% bootstrap CI, 0.134–0.294). The corresponding direct estimate was 0.164 (95% bootstrap CI, 0.040–0.283), and the total estimate was 0.372 (95% bootstrap CI, 0.256–0.481).

The indirect associations through ISI were not statistically distinguishable from zero for medical help-seeking attitudes (estimate = 0.008; 95% bootstrap CI, −0.045–0.060) or perceived neighbourhood built environment (estimate = 0.006; 95% bootstrap CI, −0.043–0.060). The estimates are presented in Table 5. These findings describe statistical indirect associations within the specified cross-sectional model and do not establish a temporal mediating process.

### 3.6. Multi-Group Comparison by Perceived Family Functioning

The ISI-derived sleep construct was positively associated with self-rated health in both the lower-functioning group (β = 0.462, *p* < 0.001) and the higher-functioning group (β = 0.241, *p* = 0.006). However, constraining the ISI–self-rated health path to equality did not significantly worsen model fit (Δχ^2^(1) = 2.502, *p* = 0.114). The analysis therefore did not provide sufficient evidence of a difference in this association between the two family-functioning groups. The multi-group comparison results are presented in Table 6.

Figure 2 summarises the structural associations, indirect estimates and family-functioning group comparison. Both statistically significant and non-significant direct paths are shown.

## 4. Discussion

This study examined psychosocial, behavioural and environmental correlates of self-rated health among 433 community-dwelling adults aged ≥40 years in Gaoping District, western China. The ISI-derived sleep construct had the largest standardised direct coefficient for self-rated health. A statistical indirect association through ISI was supported for depressive symptoms, but not for medical help-seeking attitudes or NEWS-A. The family-functioning group comparison did not identify a significant difference in the ISI–self-rated health path.

### 4.1. Sleep Quality and Self-Rated Health

The ISI–self-rated health association was consistent with evidence linking insomnia and poor sleep quality to poorer perceived health. Sleep-related difficulties may inform health appraisals through perceived symptoms and functioning, although this process was not directly examined here [3,32]. The coefficient of 0.383 describes a conditional association, not an expected benefit from improving sleep.

Interpretation remains specific to the ISI, which assesses insomnia-related difficulties rather than the broader sleep domains covered by the PSQI [33]. Comparisons with sleep-disorder prevalence estimates are also limited: 66.1% of participants were aged 40–55 years, and eligibility criteria excluded some individuals unable to participate reliably. Neither item distributions nor reverse-coded scores establish clinical prevalence.

### 4.2. Depressive Symptoms and the Indirect Association Through Sleep

Lower depressive symptom burden was associated with better self-rated health directly and through the reverse-coded ISI construct. The indirect estimate was 0.208, with a 95% bootstrap confidence interval of 0.134–0.294, while the direct association remained statistically significant. These results support the proposed statistical indirect association within the model, but do not establish that changes in depressive symptoms precede changes in sleep or perceived health.

This distinction is supported by longitudinal evidence from China. Leng et al. reported reciprocal associations between depressive symptoms and self-rated health among middle-aged and older CHARLS participants [15,34]. Their findings indicate that the relationship need not operate exclusively from depressive symptoms to perceived health. Differences in study design and measurement also preclude a direct comparison of effect magnitudes with the present study [35]. Furthermore, the PHQ-9 includes a sleep-related item. Its content overlap with the ISI may contribute to the observed association and limits mechanistic interpretation.

### 4.3. Medical Help-Seeking Attitudes and Self-Rated Health

Medical help-seeking attitudes were positively associated with self-rated health (β = 0.193), whereas the indirect estimate through ISI was small and its confidence interval included zero. Unlike the PHQ-9 findings, the results did not support the proposed sleep-related indirect association for medical help-seeking attitudes.

Research in western China links medical-resource accessibility with integrated-care demand, highlighting the relevance of healthcare context [9,36]. However, attitudes, access and service use are distinct constructs. The present findings cannot attribute the association to earlier consultation or treatment uptake, neither of which was tested. The result identifies a different association pattern from that observed for depressive symptoms.

### 4.4. Perceived Neighbourhood Built Environment and the Nonsignificant Associations

NEWS-A was positively correlated with self-rated health in the bivariate analysis, but its direct path to self-rated health was not statistically significant in the structural model. The NEWS-A–ISI path and the corresponding indirect association with self-rated health were also not supported. These results concern the conditional associations of the measured environmental construct; they do not establish that neighbourhood conditions are unimportant.

One possible explanation concerns the environmental domains assessed. The NEWS-A-based measure captured broad perceptions of access, road conditions and safety, which do not necessarily represent sleep-specific exposures [9,37]. In a longitudinal cohort study, neighbourhood night-time light and noise were associated with sleep duration trajectories. Although that study involved a different population and sleep outcome, it illustrates why broad neighbourhood perceptions and specific night-time exposures may yield different findings [15]. Measurement differences therefore offer a possible, untested explanation [38]. Shift work and commuting conditions were also absent from the model. The findings cannot establish that individual-level factors are generally more important than environmental conditions.

### 4.5. Family Functioning and the ISI–Self-Rated Health Association

Despite numerically different group-specific coefficients, the equality test did not support a difference in the ISI–self-rated health path (Δχ^2^(1) = 2.502, *p* = 0.114). This neither confirms a buffering role nor demonstrates group equivalence. Interpretation is specific to the sample-based FAD threshold of 2.00. Dichotomising continuous scores loses information and can reduce sensitivity to differences, although this cannot be established as the reason for the nonsignificant finding [4]. Measurement invariance was not established across the FAD-defined groups, which limits interpretation of the between-group path comparison.

Several limitations should be acknowledged. First, the cross-sectional design precludes causal inference and temporal ordering among depressive symptoms, insomnia-related difficulties and self-rated health. Age-related heterogeneity was not examined, and the pooled estimates should not be interpreted as demonstrating equivalent associations across age groups. Second, all constructs were assessed through self-report at a single time point, creating a risk of common method bias that may have influenced the observed associations [39]. Third, this study was conducted in one district of western China, which may limit generalisability to other settings [40,41]. Despite these limitations, this study has several strengths. It provides evidence from adults aged 40 years and older living in urban and rural communities in Gaoping District, western China, extending the geographical coverage of research on self-rated health. By examining depressive symptoms, medical help-seeking attitudes and perceived neighbourhood built environment within a common analytical framework, this study estimated their associations with self-rated health while accounting for the other specified constructs. The analysis distinguished direct associations from statistical indirect associations through the ISI-derived sleep construct and separately examined whether the ISI–self-rated health path differed between family-functioning groups. This approach provided a focused assessment of the proposed relationships, including those that were not supported in this sample.

## 5. Conclusions

Among community-dwelling adults aged 40 years and older in Gaoping District, lower depressive symptom burden and more favourable medical help-seeking attitudes were directly associated with better self-rated health. A statistical indirect association through the reverse-coded ISI construct was supported for depressive symptoms, but not for medical help-seeking attitudes or perceived neighbourhood built environment. These findings suggest that considering insomnia-related difficulties alongside depressive symptoms and help-seeking attitudes may help contextualise self-rated health in this community sample, without establishing causal mediation or intervention effectiveness.

## Figures and Tables

**Table 1:** Participant characteristics (*n* = 433).

Characteristic	Category	*n*	%
Gender	Female	260	60.0
	Male	173	40.0
Age group	40–45 years	119	27.5
	46–50 years	91	21.0
	51–55 years	76	17.6
	56–60 years	52	12.0
	61–65 years	20	4.6
	66–70 years	30	6.9
	≥71 years	45	10.4
Education	No formal education	26	6.0
	Primary school	74	17.1
	Middle school	60	13.9
	High school/ vocational/college	204	47.1
	Bachelor’s degree and above	69	15.9
Marital status	Married	387	89.4
	Unmarried	3	0.7
	Other	43	9.9
Occupation	Predominantly non-manual work	181	41.8
	Mainly physical work	59	13.6
	Retired	52	12.0
	Unemployed	83	19.2
	Both mental and physical labour	58	13.4
Economic status	Very good	25	5.8
	Good	83	19.2
	Average	297	68.6
	Poor	24	5.5
	Very poor	4	0.9

**Table 2:** Descriptive statistics and Pearson’s correlation matrix.

Construct	Mean	SD	PHQ-9	ATMHSS	NEWS-A	ISI	FAD	SRH
PHQ-9	1.63	0.60	1					
ATMHSS	2.58	0.83	0.466 **	1				
NEWS-A	3.75	0.76	0.396 **	0.527 **	1			
ISI	2.42	0.97	0.516 **	0.283 **	0.238 **	1		
FAD	1.98	0.74	0.271 **	0.321 **	0.176 **	0.282 **	1	
SRH	2.45	0.92	0.463 **	0.417 **	0.325 **	0.501 **	0.266 **	1

Note: ** *p* < 0.01. Variables are reported according to the harmonised scoring used for SEM interpretation.

**Table 3:** Structural model fit indices.

Fit Index	Recommended Criterion	Result	Assessment
CMIN	-	1697.042	-
DF	-	1066	-
CMIN/DF	<3	1.592	Good
RMR	<0.08	0.038	Good
GFI	>0.80	0.860	Acceptable
AGFI	>0.80	0.846	Acceptable
IFI	>0.90	0.956	Good
TLI	>0.90	0.953	Good
CFI	>0.90	0.955	Good
RMSEA	<0.08	0.037	Good

**Table 4:** Standardised and unstandardised path coefficients.

Structural Path	Standardised Coefficient	Unstandardised Coefficient	SE	CR	*p* Value	Interpretation
ISI ← PHQ-9	0.544	0.862	0.101	8.520	<0.001	Significant
ISI ← ATMHSS	0.020	0.023	0.070	0.324	0.746	Not significant
ISI ← NEWS-A	0.016	0.021	0.084	0.256	0.798	Not significant
SRH ← PHQ-9	0.164	0.246	0.094	2.620	0.009	Significant
SRH ← ATMHSS	0.193	0.209	0.065	3.237	0.001	Significant
SRH ← NEWS-A	0.091	0.115	0.077	1.495	0.135	Not significant
SRH ← ISI	0.383	0.363	0.056	6.490	<0.001	Significant

**Table 5:** Bootstrap mediation effects.

Path	Estimate	SE	Lower 95% CI	Upper 95% CI	*p* Value	Conclusion
PHQ-9 → SRH total effect	0.372	0.058	0.256	0.481	<0.001	Significant
PHQ-9 → ISI → SRH indirect effect	0.208	0.040	0.134	0.294	<0.001	Indirect association supported
PHQ-9→ SRH direct effect	0.164	0.061	0.040	0.283	0.009	Significant
ATMHSS→ SRH total effect	0.201	0.068	0.068	0.331	0.001	Significant
ATMHSS → ISI→ SRH indirect effect	0.008	0.026	−0.045	0.060	0.751	Not significant
ATMHSS→ SRH direct effect	0.193	0.064	0.068	0.316	0.003	Significant
NEWS-A → SRH total effect	0.097	0.061	−0.025	0.215	0.111	Not significant
NEWS-A→ ISI → SRH indirect effect	0.006	0.026	−0.043	0.060	0.776	Not significant
NEWS-A→ SRH direct effect	0.091	0.053	−0.014	0.194	0.135	Not significant

**Table 6:** Multi-group comparison of the ISI–SRH association by perceived family functioning.

Path	Low FAD Group β	C.R.	*p*	High FAD Group β	C.R.	*p*	Δχ^2^	Δdf	*p* for Group Difference
ISI → SRH	0.462	5.726	<0.001	0.241	2.771	0.006	2.502	1	0.114

Note: Lower-functioning group: directionally coded FAD scores ≤ 2.00, *n* = 265; higher-functioning group: scores > 2.00, *n* = 168. Higher scores indicated better perceived family functioning. β = standardised path coefficient; C.R. = critical ratio. The chi-square difference test compared models with the ISI–SRH path freely estimated versus constrained to equality across groups.

## Data Availability

The data presented in this study are available on request from the corresponding author due to privacy and ethical restrictions. The data are not publicly available because they contain participant-level survey information collected from human participants.

## Supplementary Materials

The following supporting information can be downloaded at: https://www.mdpi.com/article/10.3390/healthcare14203528/s1, Table S1: Measurement instruments, scoring and internal consistency; Table S2: Overall confirmatory factor analysis fit indices; Table S3: Standardised factor loadings, composite reliability and average variance extracted; Table S4: Discriminant validity assessment; Table S5: Data-screening and diagnostic statistics; Table S6: Summary of the main findings; Figure S1: Measurement model for self-rated health (SRH); Figure S2: Measurement model for depressive symptoms (PHQ-9); Figure S3: Measurement model for medical help-seeking attitudes (ATMHSS); Figure S4: Measurement model for perceived neighbourhood built environment (NEWS-A); Figure S5: Measurement model for sleep quality (ISI); Figure S6: Measurement model for family functioning (FAD); Figure S7: Overall measurement model; Figure S8: Fit indexes and parameter estimates of the structural model.

## Abbreviations

The following abbreviations are used in this manuscript:

SRH	Self-rated Health
PHQ-9	Patient Health Questionnaire-9
ATMHSS	Attitudes Toward Medical Help-Seeking Scale
NEWS-A	Neighbourhood Environment Walkability Scale-Abbreviated
ISI	Insomnia Severity Index
FAD	Family Assessment Device
