# Supplementary material for: Depressive Symptoms, Insomnia-Related Difficulties and Self-Rated Health Among Community-Dwelling Adults Aged 40 Years and Older in Western China: A Cross-Sectional Structural Equation Modelling Study
Source: Healthcare (Basel). 2026 Oct 20;14(20):3528. doi: 10.3390/healthcare14203528 (PMC13704263; doi:10.3390/healthcare14203528)
Supplement: Supplementary file 1 [file healthcare-14-03528-s001.zip › healthcare-4420195-supplementary.pdf]

Supplementary

Table S1. Measurement instruments, scoring and internal consistency.

Constructs	Measure	Items	Response scale	Direction used in SEM	Cronbach's alpha
Self-rated health	SF-36 General Health subscale	5	Item-specific response options	Higher score = better self-rated health	0.874
Depressive symptoms	Patient Health Questionnaire-9 (PHQ-9)	9	0-3	Items reverse-coded for SEM; Items reverse-coded for SEM; higher values indicate lower depressive symptom burden.	0.917
Medical help-seeking attitudes	10-item adapted medical help-seeking attitude measure (ATMHSS)	10	1-5	Reverse-coded where required; higher score = more favourable orientation toward seeking medical care	0.931
Perceived neibourhood-built environment	Neighbourhood Environment Walkability Scale-Abbreviated (NEWS-A)	17	1-5	Higher score = more favourable environmental perception	0.940
Sleep quality	Insomnia Severity Index (ISI)	7	0-4	Items reverse-coded for SEM; Items reverse-coded for SEM; higher values indicate fewer insomnia-related difficulties.	0.912

Perceived family functioning	Family Assessment Device (FAD)	12	1-4	Negatively worded items reverse-coded; higher score = better family functioning	0.935

Table S2. Overall confirmatory factor analysis fit indices.

Fit index	Value	Conventional criterion	Assessment
CMIN	2466.554	-	-
DF	1691	-	-
CMIN/DF	1.459	< 3.00	Good
RMR	0.040	< 0.08	Good
GFI	0.845	> 0.80	Acceptable
AGFI	0.832	> 0.80	Acceptable
IFI	0.955	> 0.90	Good
TLI	0.953	> 0.90	Good
CFI	0.955	> 0.90	Good
RMSEA	0.033	< 0.08	Good

CFA, confirmatory factor analysis; CMIN, chi-square; DF, degrees of freedom; RMR, root mean square residual; GFI, goodness-of-fit index; AGFI, adjusted goodness-of-fit index; IFI, incremental fit index; TLI, Tucker-Lewis index; CFI, comparative fit index; RMSEA, root mean square error of approximation.

Table S3. Standardised factor loadings, composite reliability and average variance extracted.

Variable	Measurement Indicators	Factor Loading	CR	AVE
	PHQ91	0.728		
	PHQ92	0.723		
PHQ9	PHQ93	0.764	0.918	0.554
	PHQ94	0.797		
	PHQ95	0.706		

Variable	Measurement Indicators	Factor Loading	CR	AVE
	PHQ96	0.742		
	PHQ97	0.686		
	PHQ98	0.772		
	PHQ99	0.772		
	ATMHSS 1	0.849		
	ATMHSS 2	0.849		
	ATMHSS 3	0.694		
	ATMHSS 4	0.673		
	ATMHSS 5	0.76		
ATMHSS	ATMHSS 6	0.767	0.934	0.587
	ATMHSS 7	0.758		
	ATMHSS 8	0.722		
	ATMHSS 9	0.669		
	ATMHSS 10	0.886		
	NEWSA1	0.749		
NEWSA	NEWSA2	0.814	0.86	0.605
	NEWSA3	0.786		
	NEWSA4	0.761		
	NEWSA11	0.867		
NEWSA1	NEWSA12	0.807	0.889	0.668
	NEWSA13	0.784		
	NEWSA14	0.808		
	NEWSA21	0.856		
	NEWSA22	0.893		
NEWSA2	NEWSA23	0.853	0.925	0.711
	NEWSA24	0.819		
	NEWSA25	0.791		
	NEWSA31	0.81		
	NEWSA32	0.802		
NEWSA3	NEWSA33	0.913	0.929	0.724
	NEWSA34	0.825		
	NEWSA35	0.897		
	NEWSA41	0.799		
NEWSA4	NEWSA42	0.799	0.86	0.673
	NEWSA43	0.861		
ISI	ISI1	0.761	0.912	0.598
	ISI2	0.794		

Variable	Measurement Indicators	Factor Loading	CR	AVE
	ISI3	0.732		
	ISI4	0.752		
	ISI5	0.773		
	ISI6	0.811		
	ISI7	0.789		
FAD	FAD1	0.686	0.936	0.549
	FAD2	0.721		
	FAD3	0.651		
	FAD4	0.761		
	FAD5	0.815		
	FAD6	0.775		
	FAD7	0.705		
	FAD8	0.715		
	FAD9	0.708		
	FAD10	0.747		
	FAD11	0.789		
	FAD12	0.8		
SRH	SRH1	0.737	0.875	0.584
	SRH2	0.746		
	SRH3	0.771		
	SRH4	0.769		
	SRH5	0.797		

Table S4. Discriminant validity assessment.

Construct	PHQ-9	ATMHSS	NEWS-A	ISI	FAD	SRH
PHQ-9	0.744					
ATMHSS	0.466	0.766				
NEWS-A	0.396	0.527	0.778			
ISI	0.516	0.283	0.238	0.773		
FAD	0.271	0.321	0.176	0.282	0.741	
SRH	0.463	0.417	0.325	0.501	0.266	0.764

Table S5. Data-screening and diagnostic statistics.

Diagnostic	Results	Interpretation
Questionnaires distributed	512	Questionnaires issued for the nested analytic substudy
Questionnaires returned	467	Returned questionnaires before screening
Questionnaires excluded	34	Substantial missingness, logical inconsistency, or invalid response
Analytic sample	433	Included in CFA and SEM
Multivariate outliers	None identified	Assessed using Mahalanobis distance
Item skewness	-1.217 to 2.822	No serious departure using the pre-specified descriptive criterion
Item kurtosis	-1.120 to 8.845	No serious departure using the pre-specified descriptive criterion
Tolerance	0.554 to 0.945	No substantial multicollinearity
Variance inflation factor	1.058 to 1.804	No substantial multicollinearity

Figure S1: Measurement Model for Self-Rated Health (SRH)

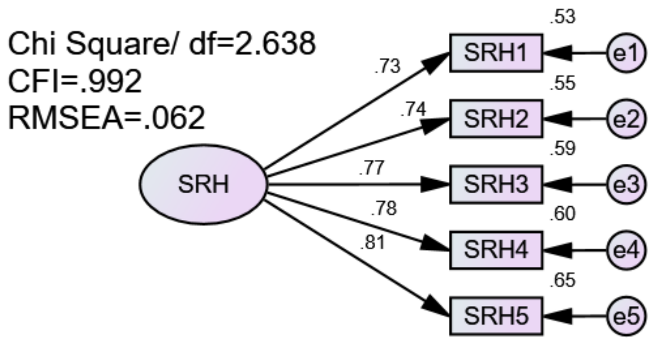

Figure S2: Measurement Model for Depressive Symptoms (PHQ-9)

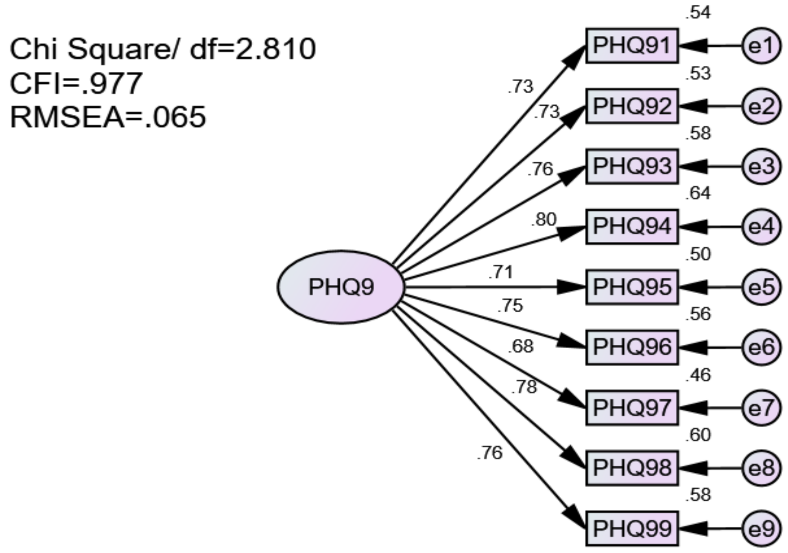

Figure S3: Measurement Model for Medical help-seeking attitudes (ATMHSS)

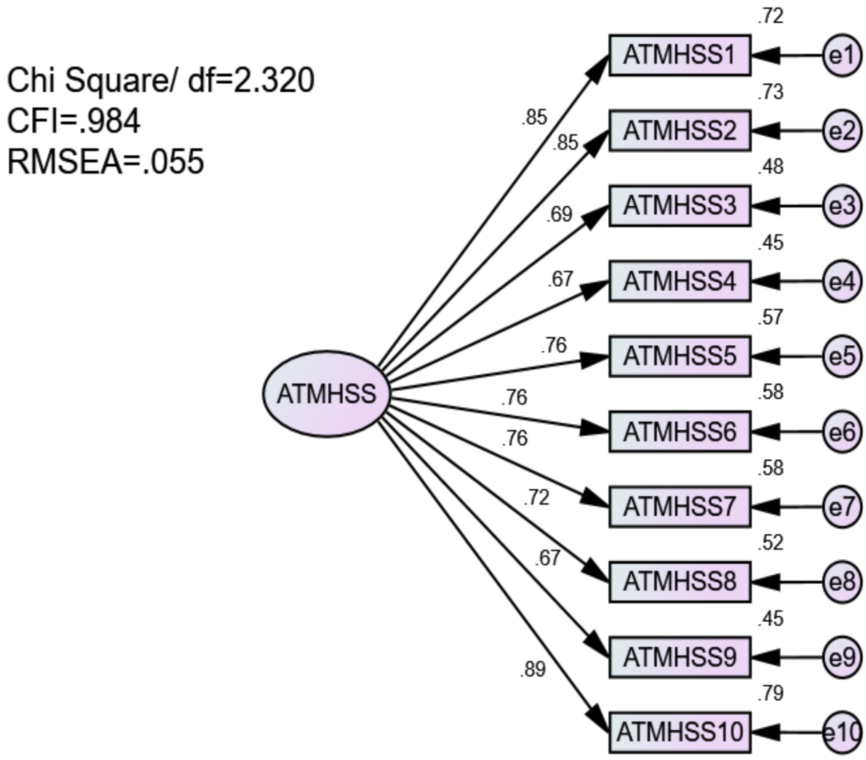

Figure S4: Measurement Model for Perceived neighbourhood-built environment (NEWS-A)

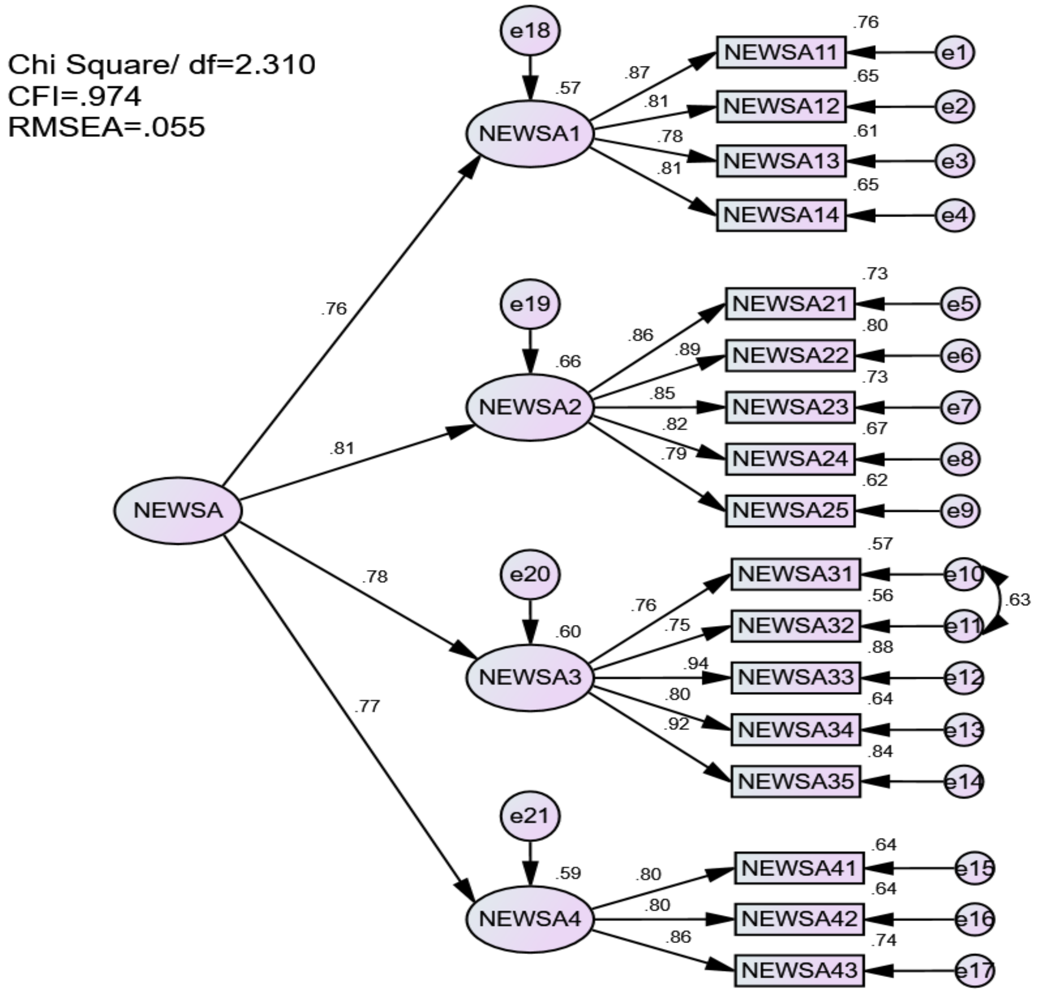

Figure S5: Measurement Model for Sleep Quality (ISI)

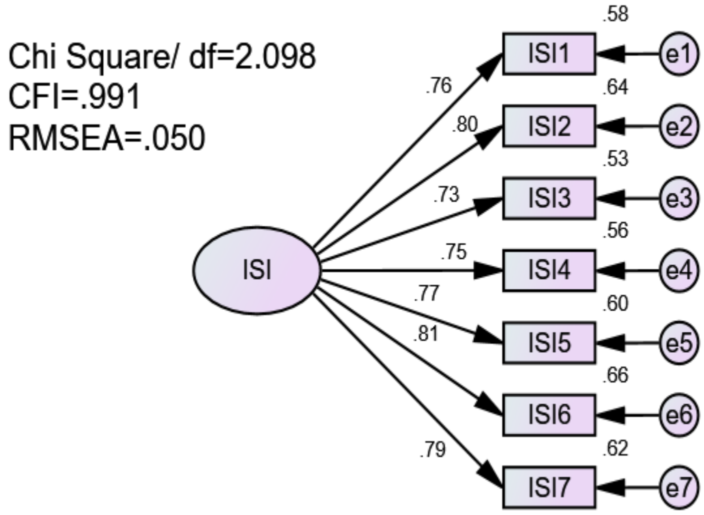

Figure S6: Measurement Model for Family Functioning (FAD)

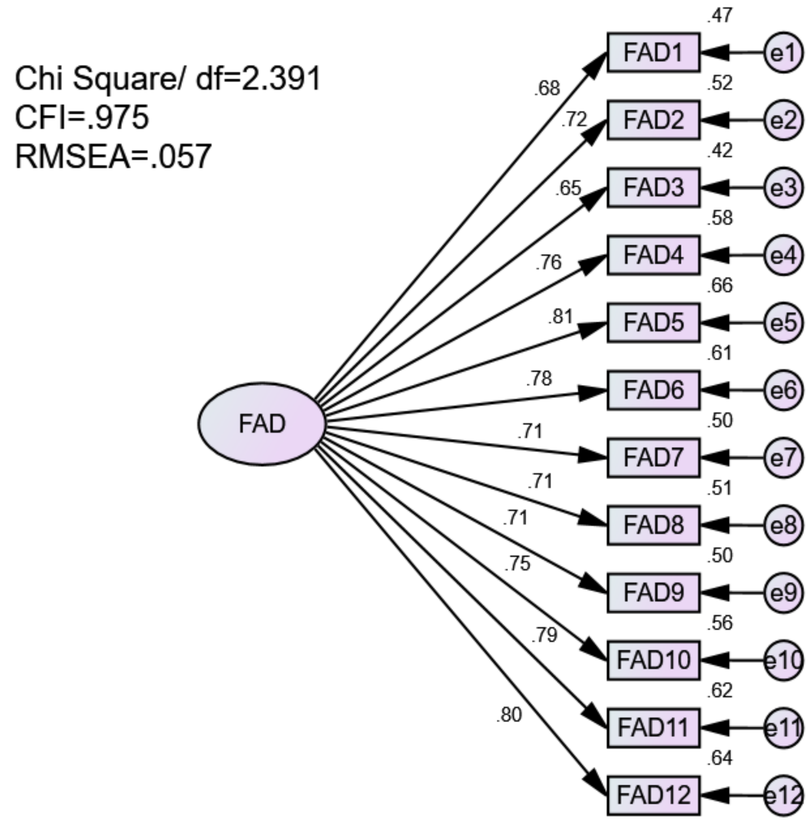

Figure S7: Overall Measurement Model

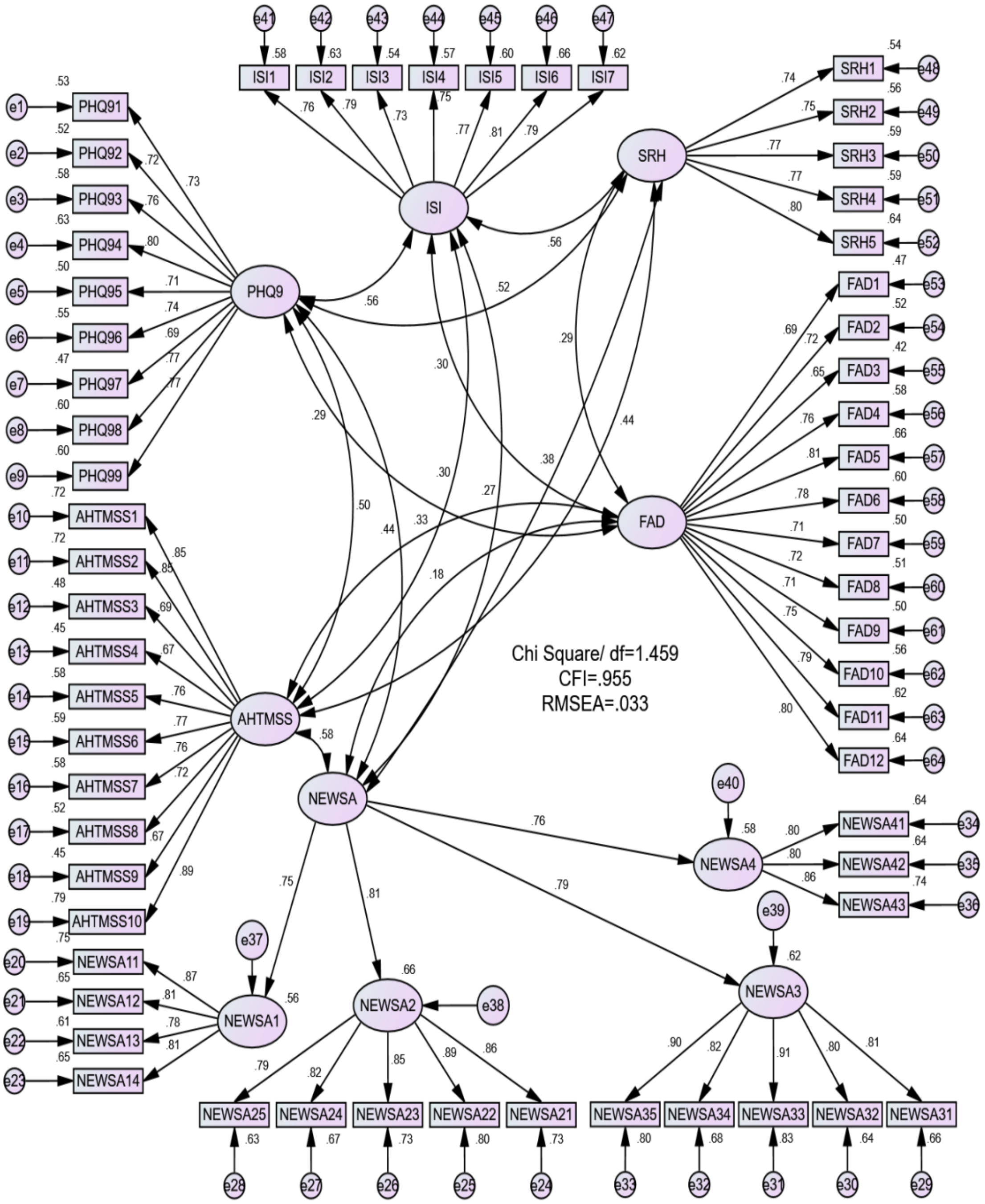

PHQ9
ISI
SRH
FAD
AHTMSS
NEWSA
NEWSA1
NEWSA2
NEWSA3
NEWSA4
Chi Square/ df=1.459
CFI=.955
RMSEA=.033

Figure S8: Fit Indexes and Parameter Estimates of the Structural Model

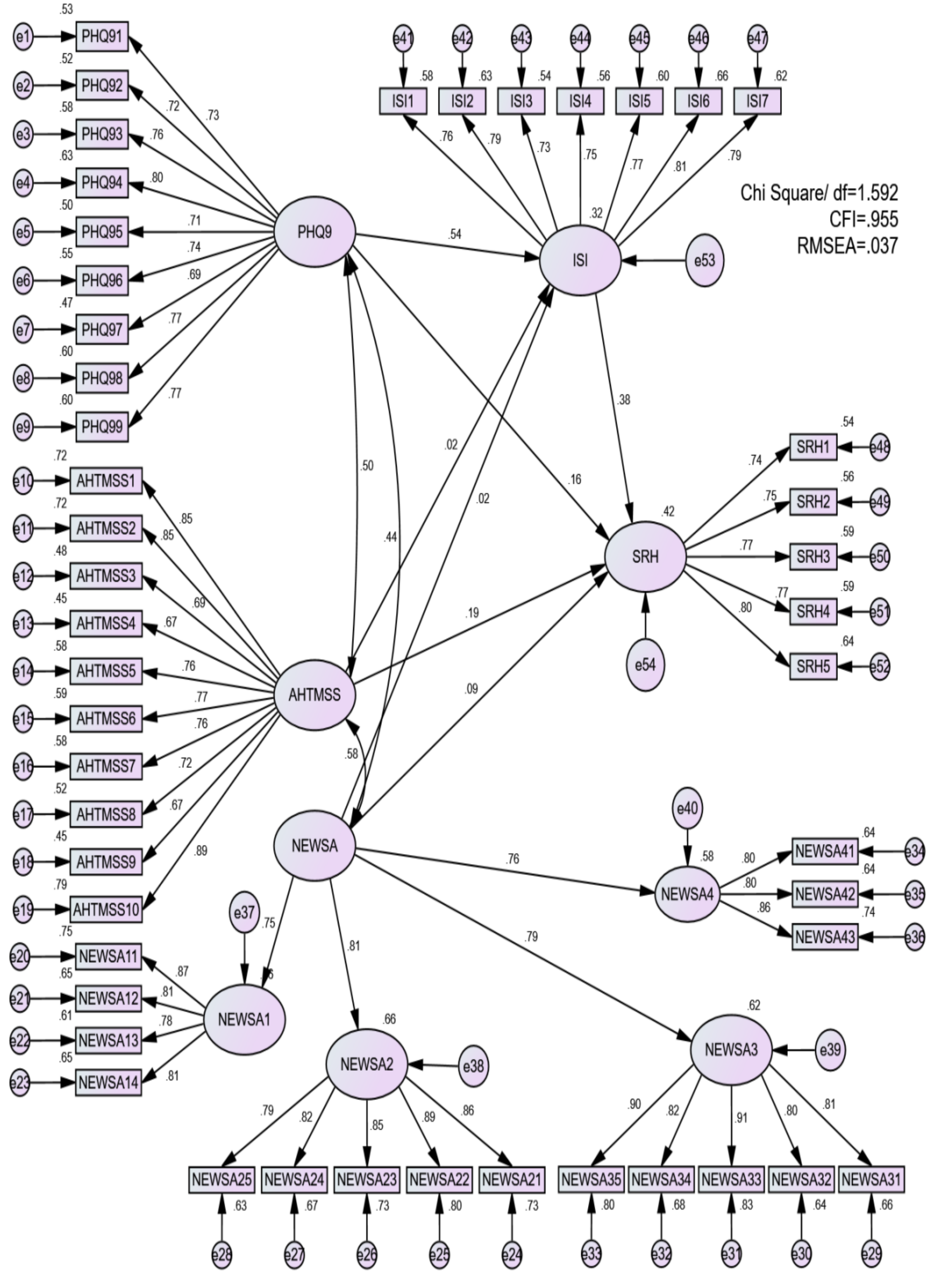

Table S6: Summary of the Main Findings

H(x)	Hypothesis	Statistical Results	Decision
**H1**	PHQ-9 → SRH	β = 0.164, p = 0.009	Supported
**H2**	ATMHSS → SRH	β = 0.193, p = 0.001	Supported
**H3**	NEWS-A →SRH	β = 0.091, p = 0.135	Not supported
**H4**	PHQ-9 → ISI	β = 0.544, p < 0.001	Supported
**H5**	ATMHSS → ISI	β = 0.020, p = 0.746	Not supported
**H6**	NEWS-A → ISI	β = 0.016, p = 0.798	Not supported
**H7**	ISI → SRH	β = 0.383, p < 0.001	Supported
**H8a**	PHQ-9 → ISI → SRH	Indirect estimate = 0.208, 95% CI [0.134, 0.294]	Supported
**H8b**	ATMHSS → ISI → SRH	Indirect estimate = 0.008, 95% CI [−0.045, 0.060]	Not supported
**H8c**	NEWS-A → ISI → SRH	Indirect estimate = 0.006, 95% CI [−0.043, 0.060]	Not supported
**H9**	FAD moderation of ISI → SRH	$\Delta\chi^2(1) = 2.502$, p = 0.114	Not supported

Note: β = standardised direct-path coefficient; CI = confidence interval; PHQ-9 = Patient Health Questionnaire-9; ATMHSS = Attitudes Toward Medical Help-Seeking Scale; NEWS-A = Neighbourhood Environment Walkability Scale–Abbreviated; ISI = Insomnia Severity Index; FAD = Family Assessment Device; SRH = self-rated health. PHQ-9 and ISI indicators were directionally coded so that higher derived scores represented a lower depressive symptom burden and better sleep quality, respectively. Direct hypotheses were classified as supported at $p < 0.05$. Statistical indirect associations were classified as supported when the 95% bootstrap confidence interval excluded zero.
